# A DNA and morphology based phylogenetic framework of the ant genus *Lasius *with hypotheses for the evolution of social parasitism and fungiculture

**DOI:** 10.1186/1471-2148-8-237

**Published:** 2008-08-19

**Authors:** Munetoshi Maruyama, Florian M Steiner, Christian Stauffer, Toshiharu Akino, Ross H Crozier, Birgit C Schlick-Steiner

**Affiliations:** 1 Department of Zoology, National Science Museum, Hyakunin-chô 3-23-1, Shinjuku-ku, Tokyo 169-0073, Japan; 2 Department of Zoology, Field Musem of Natural History, 1400 South Lake Shore Drive, Chicago IL 60605-2496, USA; 3 Institute of Zoology, Department of Integrative Biology and Biodiversity Research, Boku, University of Natural Resources and Applied Life Sciences Vienna, 1180 Vienna, Austria; 4 Institute of Forest Entomology, Forest Pathology and Forest Protection, Department of Forest and Soil Sciences, Boku, University of Natural Resources and Applied Life Sciences Vienna, 1190 Vienna, Austria; 5 School of Marine and Tropical Biology, James Cook University, Townsville, Queensland 4811, Australia; 6 Laboratory of Insect Behavior, National Institute of Agrobiological Sciences, Ôwasi 1-2, Tsukuba-shi 305-8634, Japan

## Abstract

**Background:**

Ants of the genus *Lasius *are ecologically important and an important system for evolutionary research. Progress in evolutionary research has been hindered by the lack of a well-founded phylogeny of the subgenera, with three previous attempts disagreeing. Here we employed two mitochondrial genes (*cytochrome c oxidase subunit I, 16S ribosomal RNA*), comprising 1,265 bp, together with 64 morphological characters, to recover the phylogeny of *Lasius *by Bayesian and Maximum Parsimony inference after exploration of potential causes of phylogenetic distortion. We use the resulting framework to infer evolutionary pathways for social parasitism and fungiculture.

**Results:**

We recovered two well supported major lineages. One includes *Acanthomyops*, *Austrolasius*, *Chthonolasius*, and *Lasius pallitarsis*, which we confirm to represent a seventh subgenus, the other clade contains *Dendrolasius*, and *Lasius sensu stricto*. The subgenus *Cautolasius*, displaying neither social parasitism nor fungiculture, probably belongs to the second clade, but its phylogenetic position is not resolved at the cutoff values of node support we apply. Possible causes for previous problems with reconstructing the *Lasius *phylogeny include use of other reconstruction techniques, possibly more prone to instabilities in some instances, and the inclusion of phylogenetically distorting characters.

**Conclusion:**

By establishing an updated phylogenetic framework, our study provides the basis for a later formal taxonomic revision of subgenera and for studying the evolution of various ecologically and sociobiologically relevant traits of *Lasius*, although there is need for future studies to include nuclear genes and additional samples from the Nearctic. Both social parasitism and fungiculture evolved twice in *Lasius*, once in each major lineage, which opens up new opportunities for comparative analyses. The repeated evolution of social parasitism has been established for other groups of ants, though not for temporary social parasitism as found in *Lasius*. For fungiculture, the independent emergence twice in a monophyletic group marks a novel scenario in ants. We present alternative hypotheses for the evolution of both traits, with one of each involving loss of the trait. Though less likely for both traits than later evolution without reversal, we consider reversal as sufficiently plausible to merit independent testing.

## Background

Ants of the Northern-hemispheric, temperate genus *Lasius *(Formicinae) are scientifically significant, in terms of relative abundance and ecological impact [1,2]. Because of the diversity of their signal and defense chemistry, *Lasius *ants are organisms widely used in chemical ecology [2-7] and the wide range of colony organisations makes the genus an ideal system for exploring social evolution [8-12]. Two further complex traits found in *Lasius *are yet to be adequately understood: social parasitism and fungiculture.

**Figure 1:**
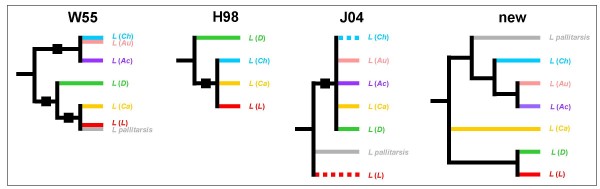
**Subgenus relationships in the previous and new phylogenetic reconstructions of the genus *Lasius***. Subgenera abbreviations: *Acanthomyops *(*Ac*), *Austrolasius *(*Au*), *Cautolasius *(*Ca*), *Chthonolasius *(*Ch*), *Dendrolasius *(*D*), *Lasius sensu stricto *(*L*). The topologies were extracted from papers by Wilson [36], "W55", Hasegawa [37], "H98", and Janda and coworkers [38], "J04", as well as from the Bayesian tree of combined, concatenated data in Fig. 2 of this paper, "new"; see Methods section for details of the procedure used for inferring the topologies W55, H98, and J04. A dotted line indicates that node support for monophyly of the subgenus was not significant. White squares indicate constraints enforced in constraint analyses using our concatenated data set in order to test the subgenus relationships of W55, H98, and J04.

Social parasitism implies that one eusocial species depends on the labour force of another [13-15]. The social parasitism exhibited in *Lasius *is temporary in that it is confined to the early stages of the parasite's colony: the parasitic queen founds her colony through entering a host colony where she kills the resident queen and takes over the worker force [1,2,13]. The study of social parasitism has become virtually a little discipline of entomology in itself [2], but the conditions for social parasitism to arise remain poorly understood [13-20]. Social parasitism has evolved many times independently in ants [2,13,15,21], but the evolutionary trajectories at finer systematic scale, e.g., whether it evolved once or multiply within genera, have only recently received detailed attention [15,18,21-27]. Fungiculture by ants, termites and beetles, on the other hand, provides a powerful study system for studying the origin and maintenance of mutualism [28]. In ants, fungiculture has evolved independently at least twice: in attines (members of the Myrmicinae), which culture the fungi for food, and in *Lasius *ants, which use fungi to build composite nest walls [29-33]. The patterns of diversification in the intensely studied attine fungiculture are only gradually starting to be understood, as brought out by recent papers on leaf-cutter fungiculture which reverse earlier impressions of certainty for some important issues [34,35]. For inferences on the evolution of the outstanding ecological and social traits including social parasitism and fungiculture in *Lasius*, a well-founded phylogeny of the genus is needed. There have been three previous studies to resolve the phylogeny of *Lasius *[36-38], but these have disagreed with each other in significant respects (Fig. 1).

In the present study we attempt to establish a robust phylogenetic framework for the relationships of the *Lasius *subgenera. We apply Bayesian analysis, a powerful tool in phylogenetic reconstruction of combined data [39] and not previously applied to *Lasius*. In addition, we also apply Maximum Parsimony analysis (MP); MP represents a completely different computational technique for phylogenetic reconstruction [40] and agreement of the reconstructions by the two independent methods would increase confidence in the tree. Our approach comprised five steps. (*i*) We combined evidence from different data sets, which for many organisms, including ants, often yields a stronger phylogenetic signal than using the data sets singly [18,39,41-50]; specifically, we combined mitochondrial DNA sequence and morphological data. (*ii*) We explored potential causes of distortion of the molecular phylogenetic signal, namely substitution saturation [51], positive selection [52,53], and compositional heterogeneity [54]. We also assessed which morphological characters may be functionally coupled with social parasitism [15,54,55], because similarities in those characters could reflect convergent adaptations to social parasitic life rather than reflect common ancestry [56,57]. We then excluded any suspected cause of distortion in the phylogenetic reconstruction. To address potential issues of character exclusion [58], we explored the effect of excluding those morphological characters suspected to be functionally coupled with social parasitism by repeating the reconstruction when including them. (*iii*) We explored whether the topologies as inferred from the different data sets are in statistically significant conflict with the phylogenetic framework inferred from the concatenated data which would tend to reduce confidence in the latter [59,60]. Our approach was to handle any conflict arising by collapsing the affected node. (*iv*) We explored whether any of the previous *Lasius *phylogenies fit our data set as well as our resulting topology. (*v*) Finally, we used posterior mapping [61,62] to define hypotheses on the evolution of social parasitism and fungiculture in *Lasius*. In all, the resulting topology provides a solid basis for studying the evolution of the various ecologically and sociobiologically relevant traits of *Lasius *across subgenera.

## Results

We found no evidence for saturation of substitutions, neither for the *16S ribosomal RNA *(*16S rRNA*) nor for any of the codon positions, single or in combination, of the *cytochrome c oxidase subunit I *(*cox1*) data. Tests for positive selection within the *cox1 *data indicated that none of the sites was subject to positive selection. But we did detect compositional heterogeneity of sequences for the third codon position of *cox1*. Recoding the nucleotides of these sites to purines and pyrimidines successfully eliminated this effect and we used the recoded sequences for all phylogenetic reconstructions. Scrutinising the morphological data set for characters potentially coupled functionally with social parasitism [1,2,13,54,55,63,64] yielded 37 characters which we hence excluded from our morphological data set. The final data set consisted of 48 samples of 30 species including three outgroup species (Table 1) for which a total of 1,265 base pairs (bp), and 64 morphological characters were used for phylogenetic reconstruction (Table 2).

**Table 1:** List of samples used for DNA sequencing and morphological analysis

Species	Subgenus	Collection locality; collector	DDBJ accession numbers		Museum voucher no
				
			*cox1*	*16S rRNA*	
*Lasius arizonicus*	*Ac.*	USA: Arizona, Madera Canyon; C.A. Schmidt	AB370982	AB371028	MMANT12
*L. interjectus*	*Ac.*	USA: Arizona, West Turkey Creek; C.A. Schmidt	AB370981	AB371027	MMANT13
*L. latipes*	*Ac.*	USA: Wisconsin, Milwaukee; J.M. Raczkowski	AB433922	AB433927	NMANT120
*L. reginae*	*Au.*	Austria: Trandorf; B.C. Schlick-Steiner & F.M. Steiner	AB370983	AB371029	MMANT23
*L. flavus*	*Ca.*	Austria: Leiser Berge; B.C. Schlick-Steiner & F.M. Steiner	AB370984	AB371030	MMANT22
*L. flavus*	*Ca.*	Russia: Ussurisky, Kaimanovka; M. Maruyama	AB370985	AB371031	MMANT38
*L. flavus*	*Ca.*	Japan: Gifu-ken, Takayama-shi, M. Maruyama	AB370986	AB371032	MMANT45
*L. nearcticus*	*Ca.*	USA: Arizona, Rustler Park; C.A. Schmidt	AB370987	AB371033	MMANT14
*L. mixtus*	*Ch.*	Austria: Göpfritz; B.C. Schlick-Steiner & F.M. Steiner	AB370988	AB371034	MMANT30
*L. umbratus*	*Ch.*	Japan: Tôkyô-to, Koganei-shi; M. Maruyama	AB370989	AB371035	MMANT6
*L. capitatus*	*D*.	Japan: Nagano-ken, Matsumoto-shi; T. Komatsu	AB370990	AB371036	MMANT44
*L. capitatus*	*D*.	Japan: Gifu-ken, Shôkawa-mura; M. Maruyama	AB370993	AB371039	MMANT47
*L. capitatus*	*D*.	Japan: Yamanashi-ken, Kitakoma-gun; M. Maruyama	AB370991	AB371037	MMANT58
*L. capitatus*	*D*.	Japan: Tochigi-ken, Haga-gun; S. Nagashima	AB370992	AB371038	MMANT62
*L. fuji*	*D*.	Japan: Hokkaidô, Maruseppu-chô; Y. Kida	AB370994	AB371040	MMANT1
*L. fuji*	*D*.	Russia: Ussurisky, Kaimanovka; M. Maruyama	AB370995	AB371041	MMANT34
*L. fuliginosus*	*D*.	Austria: Urschendorf; B.C. Schlick-Steiner & F.M. Steiner	AB370996	AB371042	MMANT24
*L. fuliginosus*	*D*.	Austria: Vienna; B.C. Schlick-Steiner & F.M. Steiner	AB370997	AB371043	MMANT70
*L. nipponensis*	*D*.	Russia: Ussursky, Vityas; M. Maruyama	AB371001	AB371047	MMANT33
*L. nipponensis*	*D*.	Japan: Hokkaidô, Sapporo-shi; M. Maruyama	AB370998	AB371044	MMANT63
*L. nipponensis*	*D*.	Japan: Nagano-ken, Fujimi-chô; M. Maruyama	AB370999	AB371045	MMANT64
*L. nipponensis*	*D*.	China: Hubei, Xianfeng; T. Kishimoto	AB371000	AB371046	MMANT67
*L. orientalis*	*D*.	Japan: Hokkaidô, Shari-chô; Y. Kida	AB371002	AB371048	MMANT4
*L. orientalis*	*D*.	Japan: Gifu-ken, Kamitakara-mura; M. Maruyama	AB371003	AB371049	MMANT60
*L. spathepus*	*D*.	Japan: Shimane-ken, Oki-shotô; T. Shimada	AB371006	AB371052	MMANT32
*L. spathepus*	*D*.	Japan: Yamanashi-ken, Nagasaka-chô; M. Maruyama	AB371005	AB371051	MMANT74
*L. spathepus*	*D*.	Japan: Kyôto-fu, Kyôto-shi; N. Fujiwara	AB371007	AB371053	MMANT77
*L. alienus*	*L*.	Austria: Braunsberg; B.C. Schlick-Steiner & F.M.Steiner	AB371008	AB371054	MMANT21
*L. austriacus*	*L*.	Austria: Feldberg; B.C. Schlick-Steiner & F.M. Steiner	AB371009	AB371055	MMANT27
*L. brunneus*	*L*.	Austria: Rassing; B.C. Schlick-Steiner & F.M. Steiner	AB371010	AB371056	MMANT25
*L. emarginatus*	*L*.	Austria: Vienna; B.C. Schlick-Steiner & F.M. Steiner	AB371011	AB371057	MMANT41
*L. hayashi*	*L*.	Japan: Gifu-ken, Kamitakara-mura; M. Maruyama	AB371013	AB371059	MMANT46
*L. hayashi*	*L*.	Japan: Chiba-ken, Kimitsu-shi; M. Maruyama	AB371012	AB371058	MMANT54
*L. japonicus*	*L*.	Japan: Kagawa-ken, Takamatsu-shi; F. Ito & Y. Ikeshita	AB371015	AB371061	MMANT19
*L. japonicus*	*L*.	Russia: Ussurisky, Kaimanovka; M. Maruyama	AB371017	AB371063	MMANT37
*L. japonicus*	*L*.	Japan: Chiba-ken, Kimitsu-shi; M. Maruyama	AB371014	AB371060	MMANT55
*L. japonicus*	*L*.	Japan: Hokkaidô, Sapporo-shi; T. Toida	AB371016	AB371062	MMANT76
*L. neglectus*	*L*.	Hungary: Budapest; B.C. Schlick-Steiner & F.M. Steiner	AB371018	AB371064	MMANT20
*L. niger*	*L*.	Austria: Vienna; B.C. Schlick-Steiner & F.M. Steiner	AB371019	AB371065	MMANT26
*L. platythorax*	*L*.	Austria: Moosbrunn; B.C. Schlick-Steiner & F.M. Steiner	AB371020	AB371066	MMANT28
*L. productus*	*L*.	Japan: Kagawa-ken, Takamatsu-shi; F. Ito & Y. Ikeshita	AB371021	AB371067	MMANT18
*L. sakagamii*	*L*.	Japan: Gifu-ken, Gifu-shi; J. Heinze	AB371022	AB371068	MMANT29
*L. sakagamii*	*L*.	Japan: Tôkyô-to, Edogawa-ku; M. Maruyama	AB371023	AB371069	MMANT56
*L*. sp.3	*L*.	Russia: Ussurisky, Kaimanovka; M. Maruyama	AB371024	AB371070	MMANT40
*L. pallitarsis*	*L pallitarsis*	USA: Arizona, Apache Ntl Forest; C.A. Schmidt	AB371025	AB371071	MMANT15
*Myrmecocystus mimicus*	n.a.	USA: California, Carrizo Plain; P.S. Ward	AB433923	AB433928	MMANT117
*Myrmecocystus mendux*	n.a.	USA: Arizona, Pima Canyon; C.A. Schmidt	AB433920	AB433925	MMANT66
*Formica japonica*	n.a.	Japan: Tôkyô-to, Shinjuku-ku; M. Maruyama	AB371026	AB371072	MMANT7

Voucher specimens have been deposited at the National Science Museum, Tokyo, with the numbers indicated and a reference to this publication (Maruyama et al. 2008/voucher no). Abbreviations of *Lasius *subgenera are *Acanthomyops *(*Ac*), *Austrolasius *(*Au*), *Cautolasius *(*Ca*), *Chthonolasius *(*Ch*), *Dendrolasius *(*D*), *Lasius sensu stricto *(*L*). Subgenus was not applicable (n.a.) for the outgroup taxa, *Myrmecocystus mimicus *and *M. mendux*, and *Formica japonica*.

**Table 2:** Character counts and substitution models for partitions

	Characters total	Characters variable but parsimony uninformative	Characters parsimony informative	AIC model selection	hLRT model selection	Model used in final MCMC runs
*cox1 *position1	281	17	22	GTR+I+Γ	GTR+Γ	GTR+I+Γ
*cox1 *position2	281	8	2	F81	F81	F81
*cox1 *position3 RY	281	28	87	-	-	F81
*16s rRNA*	422	45	83	GTR+I+Γ	GTR+Γ	GTR+I+Γ
morphology	64	10	54	-	-	Mk+Γ

RY purine + pyrimidine coding, otherwise the original nucleotide sequence was used. "-" under model selection indicates that AIC and hLRT model selection were not applicable for the partition.

**Figure 2:**
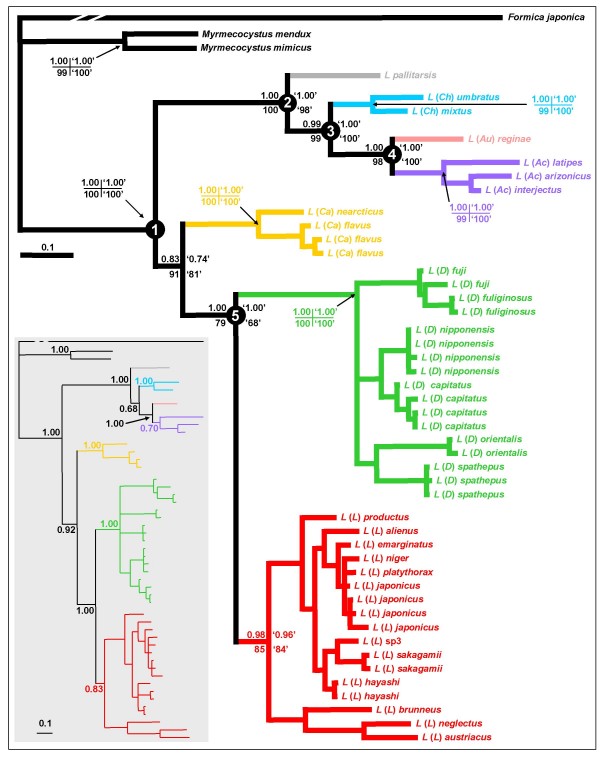
**Bayesian topology from the analysis of the combined, concatenated data**. Subgenera are abbreviated as in Fig. 1. The tree is a consensus tree resulting from a Bayesian analysis of our concatenated data set based on *cox1 *plus 16S rRNA plus morphology. The credibility values are posterior probabilities derived from 20,000 trees representing 2 million generations after burnin (upper left), bootstrap values from the 50% majority-rule consensus MP tree of the same data (lower right, in quotation marks); values for nodes following the basal divergence within subgenera are omitted. The node numbers refer to the inferred ancestral character states given in Table 5. The inset tree on grey background is a Bayesian tree based on *cox1 *plus 16S rRNA and the credibility values are posterior probabilities derived from 20,000 trees representing 2 million generations after burnin.

Topology, branch lengths, and Bayesian posterior probabilities of the Markov Chain Monte Carlo (MCMC) analyses of the concatenated data (*cox1 *plus *16S rRNA *plus morphology) are given in Fig. 2. Monophyly of all subgenera was strongly supported (0.98 – 1.00), as were all nodes defining subgenus relationships (0.99 – 1.00), with exception of one (0.83), connecting *Cautolasius *to (*Dendrolasius *+ *Lasius sensu stricto*). Strictly applying a cutoff for node support of > 0.95, we retrieved two major lineages, (((*Austrolasius *+ *Acanthomyops*) + *Chthonolasius*) + *Lasius pallitarsis*) and ((*Dendrolasius *+ *Lasius sensu stricto*), but the position of *Cautolasius *relative to these two lineages remains unresolved.

The three individual data set phylogenies (*cox1*, *16S rRNA*, morphology) differed considerably in phylogenetic resolution at subgenus level and above and none of them achieved resolution of all nodes scored by the concatenated data reconstruction. Repeating reconstruction of *cox1 *without the third codon position did not yield any well supported nodes that contradicted well supported nodes in the reconstruction using all three codon positions, which confirms the reliability of the result from the applied test for substitution saturation. Comparison of the individual data set topologies with the concatenated data topology at subgenus level and above revealed not a single significant (for MCMC, posterior probability > 0.95) disagreement but rather agreement on well supported nodes. The following nodes of the concatenated topology were supported by the individual data set topologies: *cox1 *- *Cautolasius *monophyly, *Chthonolasius *monophyly, and monophyly of the two *Myrmecocystus *outgroup species; *16S rRNA *- *Cautolasius *monophyly, (*Austrolasius *+ *Acanthomyops*), and (*Chthonolasius *+ *Lasius pallitarsis *+ (*Austrolasius *+ *Acanthomyops*)); morphology - *Dendrolasius *monophyly and (*Austrolasius *+ *Acanthomyops*). Including those morphological characters suspected to be functionally coupled with social parasitism, which we therefore had excluded before, resulted in an identical topology, with very similar node support values (Fig. 2), but with a decrease of the value for the node connecting *Cautolasius *to (*Dendrolasius *+ *Lasius sensu stricto*) from 0.83 to 0.74. MP reconstructions based on the concatenated data set significantly (node support threshold of > 70 for MP) confirmed all significant nodes of the Bayesian reconstructions, including retrieving as significant the node connecting *Cautolasius *to (*Dendrolasius *+ *Lasius sensu stricto*). This pattern persisted in the MP analysis when using the concatenated data including those morphological characters suspected to be functionally coupled with social parasitism, with one exception: the node connecting *Dendrolasius *to *Lasius sensu stricto *was then no longer supported (Fig. 2). Considering the lack of significant disagreement between the signals in the individual data set phylogenies and the confirmation of the Bayesian topology by Maximum Parsimony reconstructions, we regard the Bayesian concatenated data topology as a phylogenetic framework sufficiently robust for use in subsequent evolutionary hypotheses ("new" topology in Fig. 1).

Subsequently, we also made a Bayesian reconstruction of the concatenated molecular data sets (*cox1 *plus *16S rRNA*), without any morphological data, to allow for DNA based estimates of branch length. This tree (inset in Fig. 2) was in large agreement with the other Bayesian trees, in topology and branch lengths, but in addition to the node connecting *Cautolasius *to (*Dendrolasius *+ *Lasius sensu stricto*), three previously well supported nodes now lacked significant support (*Acanthomyops *monophyly; node connecting *Chthonolasius *to (*Austrolasius *+ *Acanthomyops*); *Lasius sensu stricto *monophyly).

In additional rounds of reconstruction, we enforced the subgenus relationships recovered in three previous phylogenetic analyses [36-38] as constraints (Fig. 1) on our concatenated data. All reconstructions with constraints had lower likelihood values than the reconstruction without constraint (Table 3) and, on the basis of our concatenated data, Bayes factor analysis revealed very strong evidence against all previously suggested subgenus relationships (Table 4).

**Table 3:** Summary of results from Bayesian analyses

Data	ngens	ln(Ar)	ln(Hr)	asdsf	burnin	99%
concatenated data: *cox1*_position 3 RY + *16S rRNA *+ morphology (Mk+Γ)	10.0	-5474.9	-5533.3	0.002	9.0	13369
W55 constraint on concatenated data	10.0	-5593.4	-5671.6	0.002	9.0	11893
H98 constraint on concatenated data	10.0	-5623.8	-5717.0	0.063	9.0	15960
J04 constraint on concatenated data	10.0	-5576.0	-5630.7	0.002	9.0	13706

ngens (number of generations) and burnin are given in units of a million; Ar and Hr refer to the arithmetic and harmonic means, averaged over the simultaneous runs; asdsf = average standard deviation of split frequencies; 99% refers to the number of trees sampled from the 99% credible set.

**Table 4:** Comparing previous Lasius phylogenies with the new phylogenetic framework

Data	Bayes factor	Interpretation
concatenated vs. W55 constraint on concatenated	276.7	very strong evidence against W55 constraint
concatenated vs. H98 constraint on concatenated	367.4	very strong evidence against H98 constraint
concatenated vs. J04 constraint on concatenated	194.9	very strong evidence against J04 constraint

Summary of Bayes factor comparisons and interpretation after [96].

The Bayesian posterior probabilities for the occurrence of social parasitism and fungiculture at the nodes of the phylogenetic framework above subgenus level ("new" topology in Fig. 1) are shown in Table 5. For eight of the ten possible inferences a state was significantly inferred (p > 0.95), for the remaining two the probability values were 0.70 and 0.82.

**Table 5:** Bayesian posterior probabilities for the occurrence of social parasitism and fungiculture as ancestral character states

Node	Social parasitism		Fungiculture	
		
	no	yes	no	yes
1	**0.98**	0.02	**1.00**	0.00
2	0.70	0.30	**0.98**	0.02
3	0.00	**1.00**	0.82	0.18
4	0.00	**1.00**	**1.00**	0.00
5	**0.97**	0.03	**0.98**	0.02

"no" indicates absence, "yes" indicates presence; the posterior probabilities were estimated using SIMMAP and the last 2000 post-burnin trees of the 20,000 used to derive the consensus tree of Fig. 2. Significantly positive values (p > 0.95) are given in bold. Node numbers refer to those shown in Fig. 2.

## Discussion and Conclusion

### The new phylogenetic framework

The new phylogenetic framework we present clarifies the relationships of all but one subgenus, namely the position of *Cautolasius *relative to the other subgenera: MP reconstructions significantly support the topology, *Cautolasius *+ (*Dendrolasius *+ *Lasius sensu stricto*), and Bayesian reconstructions do not contradict it. Given the lack of significant support in the Bayesian tree, we nevertheless subsequently refrain from considering the node as resolved ("new" topology in Fig. 1). This lack of significant resolution does not affect considerations on the evolution of social parasitism and fungiculture, though, because the ancestral state of *Lasius *was absence of both traits (Table 5), and because also *Cautolasius *displays neither trait. A significantly supported sister-status to (*Dendrolasius *+ *Lasius sensu stricto*) thus would not alter any conclusions (see discussion below). The previous phylogenies were recovered by various methods and we discuss below how the methods applied may have influenced the respective results. Taken together, the reasons for the increased robustness that we postulate for the new phylogenetic framework, which robustness is highlighted also by the confirmation of our Bayesian topology by our MP reconstructions, may be that (1) we excluded potential causes of phylogenetic distortion and (2) used additional analysis methods, that (3) the phylogenetic signal of combined data is potentially stronger than that of individual data sets [18,39,41-50], especially when applying Bayesian inference [39], that (4) all nodes but one at subgenus level or above were significantly supported in our concatenated analysis (Fig. 2), and that (5) we did not recover any disagreement between the individual data set phylogenies and the concatenated data topologies. Nevertheless, because the molecular data of this paper all derive from mitochondrial DNA, we cannot absolutely exclude that the tree is influenced by introgression at very shallow levels. Nuclear pseudogenes are also a concern, but we consider it unlikely that we amplified these because neither reading frame shifts nor sequence ambiguities were apparent. The possibilities of incomplete lineage sorting and of selection driven by symbionts which are in disequilibrium with mtDNA [65] cannot be ruled out, but such effects are unlikely to confound clade history at a deeper level, such as that of subgenera. It remains true that future studies using multiple nuclear genes are desirable to confirm our findings.

The new phylogenetic framework presented here confirms the monophyly of the six subgenera. The new framework also affirms the taxonomic placement of *Acanthomyops *as a subgenus of *Lasius *[66]. Moreover, there is additional evidence for treating *Lasius pallitarsis *as a separate, monotypic subgenus, as suggested earlier [38]. Detailed morphological characterisation of the new subgenus and taxonomic implications will be followed up elsewhere in the frame of a formal taxonomic revision.

The evolution of the *Lasius *subgenera occurred in two major lineages, the first lineage comprising *Acanthomyops*, *Austrolasius*, *Chthonolasius*, and *Lasius pallitarsis*, and the second lineage comprising *Dendrolasius*, and *Lasius sensu stricto*, with *Cautolasius *probably belonging to the second lineage (Fig. 2). Within the first lineage *Acanthomyops *and *Austrolasius *form a crown-group which is sister to *Chthonolasius*.

### Comparison with previous phylogenies

The new framework disagrees with previous topologies [36-38]. Given that we actively sought to exclude potential factors causing phylogenetic distortion from our data, and that the previous phylogenies disagreed with each other (Fig. 1), we suggest that where our topology disagrees with previous ones the new one is preferable (Table 4). Examination of the reasons for disagreements between the new topology and previous schemes is desirable and we now address this.

The phylogeny by Wilson published in 1955 [36] (W55) is the only one of the previous phylogenies that hypothesised the existence of two major lineages as recovered in the new framework. In terms of subgenera, the two W55 lineages agree with those of the new framework with the exception of *Lasius pallitarsis *(treated as *L. sitkaensis *and believed to belong to *Lasius sensu stricto *at the time [36]). However, there are disagreements within the two lineages. Within the first lineage, the situation is ambiguous because *Austrolasius*, found in the new framework to be sister to *Acanthomyops*, was treated as part of *Chthonolasius*. Within the second lineage, the relations differ in that *Lasius sensu stricto *is considered sister to *Cautolasius *in W55, whereas it is sister to *Dendrolasius *in the new framework. Reasons for the disagreements could include that W55 is based on morphological information only, and that it lacked a formal reconstruction algorithm.

In the phylogeny by Hasegawa of 1998 [37] (H98), there was only one significantly supported (i.e., for MP, node support > 70 [67]) subgenus relationship, i.e., that *Cautolasius*, *Chthonolasius*, and *Lasius sensu stricto *form a crown-group sister to *Dendrolasius*, and this is not supported by the new framework. Reasons for the disagreement could include that H98 had very limited taxon sampling, and that no check for compositional heterogeneity was undertaken: The reconstruction methods applied (Neighbour Joining and Maximum Parsimony) could also contribute as the genetic distance based Neighbour Joining reconstruction is known to have the limitation that rate variation among sites cannot be accurately accounted for [68], and as MP ignores the possible existence of a range of alternative topologies that are not significantly less likely than the most parsimonious one, even though they would require more evolutionary changes [40].

The subgenus relationships recovered by Janda and coworkers in 2004 [38] (J04), placing *Lasius sensu stricto *in a sister clade to the rest, and within this clade *Lasius pallitarsis *sister to the rest, are not supported by the new framework. However, our data confirm two important aspects of J04, the allocation of *Acanthomyops *to the genus *Lasius*, and the discovery that *Lasius pallitarsis *does not belong to *Lasius sensu stricto *or any other of the established subgenera. Reasons for the disagreements with the new framework could include that the DNA data of J04 add up to a total of only 568 bp and that no check for compositional heterogeneity was undertaken. Moreover, while the combination of molecular with other data by Janda et al. marks an advance in the history of *Lasius *phylogeny, the morphological data included 33 characters (Additional File 1) that, as indicated by other studies [1,2,12,51,52,60,61], may possibly be coupled functionally with social parasitism. Scrutiny in morphological character selection was recently confirmed as crucial in phylogenetic reconstruction [69]. Also, the occurrence of social parasitism itself was included as character. These characters might contribute to grouping all parasitic subgenera together in an unresolved crown group as indeed is the case in J04 (additionally including *Cautolasius*), whereas they were distributed over the two major lineages in the new framework. The phylogenetic reconstruction methods applied (Maximum Parsimony) could possibly have contributed to suboptimal reconstruction as compared to Bayesian inference, especially for combined data sets, demonstrated after the publication of Janda et al. [39]. Possibly none of these various causes had a strong effect *per se*, as suggested by rather minor differences between the Bayesian trees based on the concatenated data excluding *versus *including those morphological characters suspected to be coupled with social parasitism as well as between the Bayesian and the MP trees when excluding those morphological characters (Fig. 2). The various causes may, however, have added up to a significant effect, as is suggested by the lack of significant support for the sister-group relationship in the MP tree when including those morphological characters (0.68), which node scored a value of 1.0 in the Bayesian tree independently of excluding or including the characters.

### Hypotheses on the evolution of social parasitism and fungiculture

**Figure 3:**
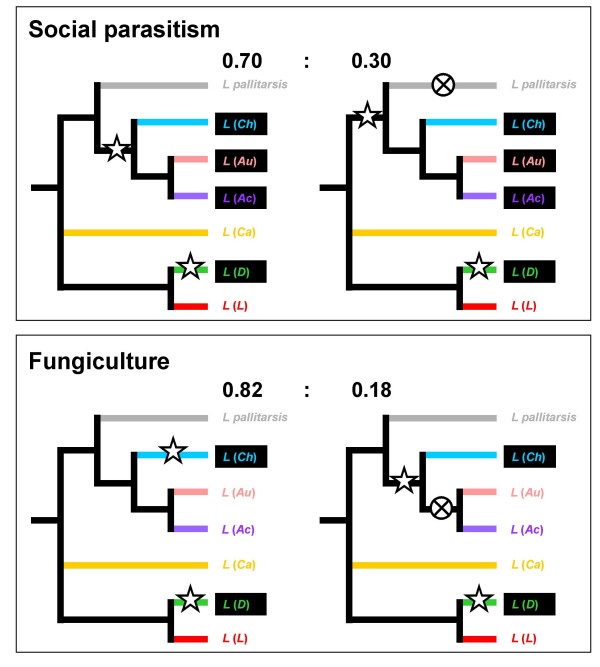
**Hypotheses on the evolution of social parasitism and fungiculture in *Lasius *ants**. Subgenera are abbreviated as in Fig. 1. Asterisks indicate the emergence of the trait, crossed circles its loss. Subgenera currently displaying social parasitism or fungiculture are indicated by frames filled black. Alternative hypotheses are offered for the evolution of social parasitism and fungiculture due to two insignificant results of the ancestral character state reconstruction in Table 5, with probabilites for the competing scenarios given, based on the values in Table 5.

The new phylogenetic framework ("new" topology in Fig. 1) and the reconstructed ancestral states (Table 5) suggest that both social parasitism and fungiculture evolved two times independently within *Lasius*, once in each of the major lineages (Fig. 3). For social parasitism such parallel evolution within a monophyletic group is not surprising in general. It has long been established that the combination of certain traits resulted in a predisposition for social parasitism in two (Myrmicinae and Formicinae) of the 21 [70] ant subfamilies [2,15,71]. Evidence that social parasitism also evolved multiple times within tribes [16,24,72] or within genera [15,21,22,25,27] confirms the principle for lower taxonomic levels. Having a reliable phylogenetic framework for *Lasius *facilitates examination of temporary social parasitism, considered less derived than other types of social parasitism [13,15,24]. One factor that may have favoured the rise of parasitism in *Lasius *may derive from their colony organisation: In several species of the non-parasitic subgenera, *Lasius sensu stricto *and *Cautolasius*, the lack of aggression between different single-queened colonies has been reported [8,17,73,74]. Whereas this behaviour concerns intraspecific interactions, the predisposition to reduce aggression may have been important for social parasitism to arise. The paramount significance of reduced aggression is illustrated by the chemical disguise of founding queens of *Chthonolasius *to appease host workers [1,2] and the exceptional winter activity of *L*. (*Ch*.) *mixtus *to ease entry to the then less aggressive host colonies [1]. Characterisation of such potentially preadaptive traits and search for them in extant non-parasitic *Lasius *species might help in finding potential early stages of incipient social parasitism. Such discoveries would then contribute to resolving mechanisms in the evolution of social parasitism in general [14-17]. Fungiculture on the other hand only is known from one other group of ants, the Attini (Myrmicinae). It is established that fungiculture arose only once in attines [35,75] and the *Lasius *situation might thus indicate a stronger predisposition to evolve fungiculture for ants generally.

For the evolution of both fungiculture and social parasites two pairs of alternative hypotheses remain (Fig. 3), both concerning the lineage of (((*Lasius pallitarsis *+ ((*Chthonolasius *+ (*Austrolasius *+ *Acanthomyops*))). This is because for both traits the probability value for the ancestral state for one node was below 0.95 (Table 5). For each trait there is one scenario considering an earlier origin with subsequent loss in the respective lineage, and the other considering an origin at a later stage in the respective lineage. Concerning both traits the hypothesis of a reversal is less likely from the point of view of biological realism because it is less parsimonious, and also because the probability values for the respective nodes (2 and 3) indicate, though not significantly, that social parasitism and fungiculture had not yet evolved at those stages (0.70 and 0.82). Still, we cannot formally refute the reversal scenarios here, given the lack of statistical support, and briefly discuss them in the below.

For social parasitism, a reversal has been considered generally unlikely [2,17]. On the other hand, reversal from temporary social parasitism may be more likely than reversal from any other, more derived type of social parasitism. For example, temporarily socially parasitic ant species of *Formica sensu stricto *that are capable of alternative nest foundation through colony budding or adoption into another colony show a certain flexibility in the nest foundation mode in this type of social parasitism [1]. On the other hand, the fact that *Acanthomyops*, *Austrolasius*, and *Chthonolasius *form an entirely socially parasitic clade shows that temporary social parasitism may in fact be a stable trait and makes reversal appear less likely.

Once arisen, fungiculture is not known to have been lost in insects and loss has been regarded as improbable [28], which provides an additional argument against the scenario involving a loss in *Lasius*. It may, however, be more probable in the *Lasius *case: fungiculture for nest building is probably less likely to entail a high dependence of the ants as compared to fungiculture for nutrition, which involves physiological adaptations [75].

It is not possible to definitely decide in favour of any of the alternative hypotheses at present, neither concerning social parasitism nor fungiculture, but it is worth considering the question further because a plausible loss of either would be of considerable evolutionary significance. For both traits the fossil record may offer answers in combination with a sound molecular clock for *Lasius*, for social parasitism by revealing the queen morphology of the ancestor of the entire major lineage, for fungiculture by a preserved fungal nest structure. Laboratory experiments may also be helpful for the analysis of both traits, for social parasitism by checking whether any of the temporary social parasites of the lineage are capable of independent colony foundation, and for fungiculture whether *Chthonolasius *colonies can be maintained when deprived of their fungus.

This study provides a basis for studying the evolution of the various ecologically and sociobiologically relevant traits across the ca. 100 [70] species of *Lasius*, by establishing a phylogenetic framework and resolving the position of six of the seven lineages at subgenus level. We have used the framework to define hypotheses of the evolution of two outstanding traits, social parasitism and fungiculture, the evolution of which continues to pose riddles to evolutionary biology. Our results suggest that both traits arose twice in *Lasius *which opens up new opportunities for comparative analyses in a close phylogenetic relationship. We present competitive hypotheses that either do or do not involve reversal from the traits.

## Methods

### The study system

The species of the ant genus *Lasius *are currently placed in six subgenera, *Acanthomyops*, *Austrolasius*, *Cautolasius*, *Chthonolasius*, *Dendrolasius*, and *Lasius sensu stricto *[70]. The most recent taxonomic revision at the genus level dates back to 1955 [36]. It recognised four subgenera: *Cautolasius*, *Chthonolasius*, *Dendrolasius*, and *Lasius sensu stricto*. The fifth subgenus, *Austrolasius*, was established in 1967 [70] and includes one species which had up to then been placed in *Chthonolasius*. The sixth subgenus, *Acanthomyops*, has an unstable history in that its status changed several times between that of a separate genus and being a subgenus, mostly of *Lasius *[70]. Evidence for its inclusion into *Lasius *has accumulated [38,76] and it was therefore formally returned to *Lasius *[66]. Monophyly of the subgenera is supported by various pieces of evidence [77], except for *Lasius sensu stricto*. *Lasius sensu stricto *harbours one taxon, *L. pallitarsis*, which on morphological and molecular grounds has been hypothesised to best constitute a separate subgenus [38]. We here validate these earlier findings [38].

Social parasitism is confined to four subgenera, *Acanthomyops*, *Austrolasius*, *Chthonolasius*, and *Dendrolasius*, with all species of these subgenera obligatorily displaying this lifestyle [1,66]. One subgenus, *Dendrolasius*, is hyperparasitic in that it parasitises parasitic *Chthonolasius *species [1,36]. Fungiculture is known from two subgenera, *Chthonolasius*, and *Dendrolasius *[34] and, as far as known, all members of the two subgenera use fungi (B.C. Schlick-Steiner, unpublished data; M. Maruyama, unpublished data; [1]), although New World species still await study.

### Taxon sampling

The material of this study is listed in Table 1. It comprises 27 species of *Lasius *(including one undescribed) representing all 6 of the subgenera currently recognised as well as *Lasius pallitarsis*. We thus present data on more than a quarter of the currently 100 valid extant *Lasius *species [70], covering both the Palaearctic and the Nearctic regions. The number of species per subgenus ranged from one to 11. Only two and three species each of *Chthonolasius *and *Acanthomyops*, respectively, were analysed, with each sample carefully chosen to be unambiguously identified to species (because the species of these subgenera are known for their habitual multidirectional hybridisation [78,79] which is likely to compromise the resolving power of gene sequences [80] and distorts morphological characters [79]). To account for intraspecific diversity, up to four colonies per species were included wherever possible. Two species of *Myrmecocystus*, which genus is sister to *Lasius *[[42,49]; confirmed by a personal communication by P.S. Ward, of February 2008], as well as *Formica japonica *were used as outgroup. Species were identified according to [1,36,77,81-83]. Voucher specimens are deposited at the National Science Museum, Tokyo under the voucher numbers listed in Table 1.

### Molecular protocols

Some samples were irreplaceable dried museum specimens. Therefore, genomic DNA was extracted from whole body using a DNAeasy tissue kit (Qiagen, Hilden, Germany) using established protocols [84] without any damage to the voucher specimens. We used 1 μl of DNA (25 – 50 ng/μl) as template for PCR amplification. A 490 – 550 bp region of *16S rRNA *was amplified and sequenced using primers "16Sar-L" 5'-CGCCTGTTTATCAAAAACAT-3' and "16Sar-L2" 5'-CCGGTCTGAACTCAGATCATG-3' originally taken from [85] but the latter one slightly altered and thus renamed. A ca. 900 bp region of *cox1 *was amplified and sequenced using the new, degenerated primers "Lasius-L" 5'-TAYCCGCCATTAGCTTCAAA-3' and "Lasius-R" 5'-TGAAATTAAGGATCCAATWGA-3'. Reactions were carried out at 10 μl volumes in a PCR Thermal Cycler MP (TaKaRa Bio Inc.) under the following conditions: a first cycle of 94°C for 3 min, followed by 35 cycles of 94°C for 30 s, annealing at 50°C for 50 s, and finally 72°C for 1 min for the *16S rRNA*; for *cox1 *all settings were identical except for annealing which was set to 42°C for 1 min 15 s. PCR products were purified with 0.5 μl of ExoSap-IT (GE Healthcare Life Sciences). All products were sequenced in both directions using BigDye Terminator v3.1 (Applied Biosystems) on an ABI 3100 Avant DNA Sequencer (Applied Biosystems) at the National Science Museum, Tokyo. The sequence data were deposited at DNA Data Base of Japan, DDBJ (see Table 1 for accession numbers).

### Exploration of molecular data concerning potential causes for phylogenetic distortion

The *16S rRNA *and *cox1 *sequences were aligned with default settings of the program Clustal X v1.83 [86]; ambiguously aligned sites in the *16S rRNA *alignment were excluded. We partitioned the *cox1 *sequences into the first, second and third codon positions using the program DAMBE v4.2.13 [87]. This program was also used to perform tests for the saturation of substitutions [88] on the *cox1 *and *16S rRNA *data. For *cox1 *all codon positions were tested simultaneously, as well as separately. We found no indication of substitution saturation (see Results for detail), but we additionally performed the *cox1 *MCMC analysis without the third codon position.

To detect potential positive selection we used the program HYPHY [89] accessed through the Datamonkey interface http://www.datamonkey.org. Mean numbers of nonsynonymous substitutions (dN) and synonymous substitutions (dS) per site (ratio dN/dS) were estimated in the *cox1 *data using the fixed effect (two-rate FEL) method and basing estimates on a Neighbour-Joining tree under the HKY substitution model. We used a nominal alpha level of 0.1.

To avoid any effect from compositional heterogeneity of sequences [90] on the phylogenetic reconstruction we separately tested each codon position of *cox1 *as well as *16S rRNA *using the program TREE-PUZZLE 5.2 [91]. When we found indications for compositional heterogeneity we recoded the sequences into purines and pyrimidines and repeated the test.

### Morphological analysis

The morphological characters considered are presented in Additional File 1. Our rationale in composing the character set was aiming at, first, a comprehensive capturing of variation at the species, subgenus and genus level, and, second, exclusion of any characters potentially distorting the phylogenetic reconstructions. We started from the complete set of character definitions of Janda et al. [38]. Pursuing our first aim, we applied 67 characters and their states exactly as described by [38], adapted – because analysis of our material revealed the necessity to do so – the definitions of character states, partly also concerning the number of character states, for another 16 characters, out of which 7 characters also were adapted in the character definitions themselves, and added 20 entirely new characters. Pursuing our second aim, we excluded from the set presented by [38] the three behavioural/ecological characters (among others the occurrence of social parasitism), and on the basis of information from [1,2,13,54,55,63,64] we further excluded 37 morphological characters, including four of the new ones. The excluded characters were characters which we suspected to be functionally coupled with temporary social parasitism, either (*i*) because of direct functional reasoning, or (*ii*) because they are known to be correlated with social parasitism in other ant genera which contain both parasitic and non-parasitic species. The characters excluded under (*i*) concerned the mandible, which is needed by parasitic *Lasius *queens to dismember host workers and strangle the host queen; the maxillary palp and the scape, which may be under selective pressure to be short and robust so as to escape damage by aggressive hosts in the initial stage of colony take-over; and the mesosoma size, because parasitic, i.e., dependently founding, queens need less tissue storage than independently founding queens. The characters excluded under (*ii*) concerned the length of hairs, which can be either extremely long or extremely short in parasites; the pubescence, which is absent in some parasites; the size of the head and the overall body, which both are frequently small in parasitic queens; and the shape of the petiole, which is frequently aberrant in parasites. We finally also excluded three characters for which we observed no variation in our material (their general lack of variation for the species analysed confirmed by a personal communication by B. Seifert, of April 2008). For details of how we composed our morphological data set including information on the provenance of characters and whether we excluded them, see Additional File 1. Overall, we included 64 morphological characters in our reconstructions; 35 of these concern adult workers, 18 adult queens, 23 adult males, and 2 worker larvae; 47 are binary and 17 are multi-state. To explore the effect of excluding characters, as we had done, on phylogenetic reconstructions, we also subjected the complete data set to reconstructions (but excluding invariant characters); this data set included 99 characters. We assessed any morphological data by analysis of voucher material housed in the National Science Museum, Tokyo, except the three characters concerning larval morphology. A total of 155 specimens were analysed. All multistate characters were treated as unordered.

### Phylogenetic reconstructions

To select the best-fitting nucleotide substitution models for *cox1 *and *16S rRNA *we used the hierarchical likelihood ratio test (hLRT) and the Akaike Information Criterion (AIC) implemented in the program MrModeltest 2.2 [92]. When sequence partitions had been recoded into purines and pyrimidines, models were adjusted to account for the two state character of the data. AIC and hLRT in one instance differed in the selection of models for the single DNA sequence data partitions (Table 2). AIC selected the more parameter rich model and we opted for this solution as erring on the side of overparameterisation is preferable over the opposite [93,94].

For the morphological data the Markov *k *(Mk) model [95] was applied both with (+Γ) and without gamma-distributed rates of character change in separate MCMC runs. We used Bayes factors for model selection as they are established to provide good orientation tools in this [39] and calculated them as follows, using the outcomes of the single MCMC runs: 2LnB_10 _= 2 × (Harmonic Mean Ln likelihood for Mk – Harmonic Mean Ln likelihood for Mk+Γ). In the interpretation of the yielded absolute value of 2.5 in favour of the Mk+Γ model we followed published recommendations [96], on page 777, and consequently used the Mk+Γ model for all reconstructions.

Bayesian analysis using MCMC was performed with MrBayes 3.1.2 [97] on the individual data sets (*cox1*, *16S rRNA*, morphology) and the combined, concatenated data set (*cox1 *plus *16S rRNA *plus morphology). We also analysed the combined, concatenated data set including those morphological characters suspected to be coupled with social parasitism. In addition, we analysed the concatenated molecular data (*cox1 *plus *16S rRNA*) without any morphological data. Data partitions were established to allow model parameters to be separately estimated for all partitions and additionally for the single codon positions of *cox1*. 10,000,000 generations with a sample frequency set to 100 were run. As after 9,000,000 generations stationarity was achieved with average standard deviation of split frequencies in all cases constantly below 0.002 except the reconstruction with the W55 constraint for which it was 0.063 (Table 3), we always used the last 10,000 trees of each run to compute a majority rule consensus tree assigning posterior probabilities of tree topology. We also confirmed that true convergence had been reached and that the MCMC was sampling from the posterior distribution by repeating all runs three times and checking for congruence across the runs. All runs were performed using parallel versions of MrBayes, implemented on a SGI Origin 3800 under IRIX version 6.21m, of HPC, James Cook University. All MCMC runs achieved stationarity and detailed statistics on the runs are presented in Table 3. In the interpretation of the MCMC trees we followed previous authors [94,98,99] to regard only nodes with node support of p > 0.95 as significantly supported in Bayesian analysis. We also applied this cutoff when comparing the MCMC trees based on the individual data sets and that based on the concatenated data.

We also performed MP analysis of the combined, concatenated data, as well as of the combined concatenated data adding those morphological characters suspected to be coupled with social parasitism. All MP analyses were unweighted and performed with PAUP* 4.0b10 [100] using the heuristic search algorithm with tree bisection reconnection branch swapping and 10 random stepwise additions. All characters were treated as unordered and polymorphic states were taken into account. Node support was calculated by 1,000 bootstrap replicates. In the interpretation of the MP trees, we applied the widely accepted node support threshold of > 70 [67].

We deposited the aligned, concatenated data matrix with TreeBase (Study accession number S2136).

### Comparison with previous *Lasius *phylogenies

To compare the subgenus relationships of W55, H98 and J04 directly with the new, Bayesian framework, we enforced the various topologies as constraints (Fig. 1) on our concatenated data. We then performed additional MCMC runs of our concatenated data under these constraints and compared the outcomes of the single MCMC runs using Bayes factors as given under morphological analyses. In extracting the topologies from the literature we proceeded as follows. For W55 we used the tree of "Fig. 2" of [36] slightly modified. We adopted the position of *Acanthomyops*, explicitly treated as ingroup of *Lasius *in the tree, although Wilson did not formally treat *Acanthomyop*s as a subgenus of *Lasius *in the taxonomic revision itself. We allocated *Lasius sitkaensis*, now treated as a junior synonym of *L. pallitarsis*, to *Lasius sensu stricto*. A similar situation pertains to *Austrolasius*: the subgenus had not yet been established in 1955, but the then only known species which today is treated under *Austrolasius*, *L. carniolicus*, was allocated to *Chthonolasius *and we accounted for this in our treatment of W55. For H98 we applied the node support threshold of > 70 [67] to the Maximum Parsimony reconstruction presented in "Fig. 1" of [37], and we applied the same threshold for J04, to the Maximum Parsimony reconstruction presented in "Fig. 6" of [38].

### Posterior mapping analysis

To estimate the probabilities of the possible ancestral states at each well supported node of the concatenated Bayesian topology we chose the Bayesian approach of posterior mapping [61,62], using the program SIMMAP 1.0 [101] freely available online http://www.SIMMAP.com. In contrast to parsimony approaches to character mapping this is a probabilistic approach, which (*i*) does not assume that only a single change has occurred along any branch, and (*ii*) is not prone to underestimation of the variance in ancestral state assignments [101]. Further, the SIMMAP approach allows uncertainty in phylogenetic reconstruction. A single stochastic mapping was done per tree using the last 2,000 post-burnin trees of the 20,000 trees used to derive the consensus tree of Fig. 2 and the ancestral states were inferred for the consensus tree from the MrBayes analysis.

## Authors' contributions

MM initiated the study, participated in its design and coordination, carried out the DNA sequencing work, performed the sequence alignment, and sampled the morphological characters. FMS participated in the design and coordination of the study, in the data analysis, in drafting a first version of the manuscript, and in revising it. TA helped with the design of the study. CS helped with the design of the study. RHC helped to design the data analysis and the format of the manuscript. BCS–S participated in the design and coordination of the study, in the data analysis, in drafting a first version of the manuscript, and in revising it. All authors read and approved the final version.

## Supplementary Material

Additional File 1 **Definition and data matrix of morphological characters**. L worker larva, M adult male, Q adult queen, W adult worker. The characters are consecutively numbered in the definitions which equal the numbers used in the matrix. The numeric codes for the character state definitions are given in parentheses. If a species is polymorphic in character states, both states are given in parantheses. In the penultimate row, the corresponding character name of Janda et al. [38] is given and the following signs describe the relation to [38] concerning the definitions, "*" indicates that character and states are identical, "**" indicates that the characters are identical but the states have been adapted, "***" that characters and states have been adapted, and "****" indicates new characters, not used in [38]. The letters in ultimate row indicate: "a" character used for reconstruction of new phylogenetic framework, "b" character suspected to be functionally coupled with social parasitism or a behavioural/ecological character, "c" invariant character. The samples of the matrix are identical with those given in Table 1. Subgenera are abbreviated as in Table 1. Click here for file
